# Ubiquitin C-terminal hydrolase isozyme L1 is associated with shelterin complex at interstitial telomeric sites

**DOI:** 10.1186/s13072-017-0160-2

**Published:** 2017-11-10

**Authors:** Aleksandar Ilic, Sumin Lu, Vikram Bhatia, Farhana Begum, Thomas Klonisch, Prasoon Agarwal, Wayne Xu, James R. Davie

**Affiliations:** 1 Children’s Hospital Research Institute of Manitoba, University of Manitoba, 715 McDermot Avenue, Room 600A, Winnipeg, MB R3E 3P4 Canada; 2 Department of Human Anatomy and Cell Science, University of Manitoba, 130-745 Bannatyne Ave, Winnipeg, MB R3E 0J9 Canada

**Keywords:** UCHL1, Shelterin complex, Interstitial telomeric sites, Prostate cancer

## Abstract

**Background:**

Ubiquitin C-terminal hydrolase isozyme L1 (UCHL1) is primarily expressed in neuronal cells and neuroendocrine cells and has been associated with various diseases, including many cancers. It is a multifunctional protein involved in deubiquitination, ubiquitination and ubiquitin homeostasis, but its specific roles are disputed and still generally undetermined.

**Results:**

Herein, we demonstrate that UCHL1 is associated with genomic DNA in certain prostate cancer cell lines, including DU 145 cells derived from a brain metastatic site, and in HEK293T embryonic kidney cells with a neuronal lineage. Chromatin immunoprecipitation and sequencing revealed that UCHL1 localizes to TTAGGG repeats at telomeres and interstitial telomeric sequences, as do TRF1 and TRF2, components of the shelterin complex. A weak or transient interaction between UCHL1 and the shelterin complex was confirmed by immunoprecipitation and proximity ligation assays. UCHL1 and RAP1, also known as TERF2IP and a component of the shelterin complex, were bound to the nuclear scaffold.

**Conclusions:**

We demonstrated a novel feature of UCHL1 in binding telomeres and interstitial telomeric sites.

**Electronic supplementary material:**

The online version of this article (10.1186/s13072-017-0160-2) contains supplementary material, which is available to authorized users.

## Background

Ubiquitin C-terminal hydrolase isozyme L1 (UCHL1) is a highly conserved protein, abundant in neurons—making up 1–2% of total brain protein—and present at lower concentrations in neuroendocrine cells [1]. Despite the fact that UCHL1 has been linked with neurodegenerative diseases and a wide range of cancers, its physiological role remains elusive [2]. UCHL1 hydrolyzes small C-terminal adducts of ubiquitin to generate free monomeric ubiquitin. However, its activity is low compared to that of other deubiquitinating enzymes, and whether it is a true hydrolase enzyme is a matter of debate [1, 3–5]. Conversely, UCHL1 was shown to protect the protein NOXA (also called PMAIP1) from proteasomal degradation by removing K48-linked polyubiquitin chains [6]. Similarly, p53, HIF-1α and EGFR are thought to escape turnover as a result of UCHL1 hydrolase activity [7–9]. In vitro studies have suggested that UCHL1 has a dimerization-dependent ubiquitin ligase function [10]. This ubiquitination function was reported to cause the destabilizing effect of UCHL1 on SMN and MDM2 proteins [9, 11]. In neurons, UCHL1 may affect ubiquitin homeostasis by stabilizing monoubiquitin, and this role is independent of UCHL1 hydrolase or ligase functions [12].

Although UCHL1 is strongly expressed in neurons, it is also present at high levels in many non-neuronal tumors, implicating that UCHL1 is an oncogene. However, this is not generally applicable as the UCHL1 gene can be silenced via promoter hypermethylation in some tumors, suggesting a potential suppressor function for UCHL1 in certain tumors [13]. In the case of prostate cancer, comparative proteomic data from prostate cancer cell lines showed that UCHL1 was expressed in androgen insensitive cell lines [14]. Furthermore, UCHL1 knockdown studies on the androgen insensitive DU 145 cell line demonstrated that UCHL1 promotes prostate cancer metastasis by the induction of epithelial-to-mesenchymal transition (EMT) [15]. Knockdown of UCHL1 in HEK293T cells attenuated the expression of genes involved in proliferation and migration and increased expression of genes involved in apoptosis and cell cycle arrest [16].

In this study, we analyzed the proteins cross-linked to nuclear DNA by cisplatin in situ in a panel of human prostate cancer cell lines derived from benign hyperplasia or various metastatic sites. We identified UCHL1 as a protein cross-linked to DNA in some of the prostate cancer cells, including DU 145, derived from brain metastasis. To explore the UCHL1 role, we examined its genomic distribution and its association with proteins binding the same DNA sequence motif or DNA structure.

## Results

### Identification of UCHL1 as a DNA-associated protein

**Fig. 1:**
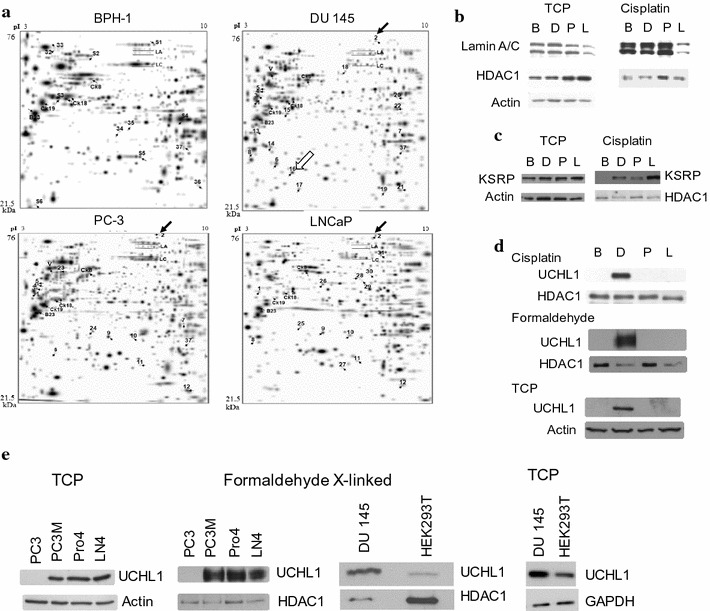
UCHL1 is associated with genomic DNA in prostate cancer cells expressing it. **a** DNA cross-linked proteins from BPH-1, DU 145, PC-3 and LNCaP cells treated with 1 mM cisplatin were electrophoretically resolved on two-dimensional PAGE. The gels were stained with silver. **b**–**e** Total cellular proteins (TCP) or DNA cross-linked proteins isolated by hydroxyapatite column chromatography from cells treated with 1 mM cisplatin or 1% formaldehyde were resolved by SDS-10% PAGE and immunoblotted with indicated antibodies. Cells in **b**–**d** were BPH-1 (*B*), DU 145 (*D*), PC-3 (*P*) and LNCaP (*L*)

To identify proteins differentially bound to genomic DNA in human prostate cancer cell lines, we compared the two-dimensional gel patterns of proteins cross-linked to genomic DNA with cisplatin. The prostate cell lines under study were BPH-1 from benign hyperplasia, androgen receptor-positive LNCaP derived from a metastatic lymph node site, androgen receptor-negative DU 145 and PC-3 derived from metastatic brain and bone sites, respectively. Proteins cross-linked to DNA in cells with cisplatin were captured on hydroxyapatite. Protein–DNA cross-links were reversed with thiourea, and the proteins were isolated and resolved by two-dimensional polyacrylamide gel electrophoresis (PAGE) (Fig. 1a). We identified the lamins A/C as proteins cross-linked by cisplatin in the four cell lines (Fig. 1b). The identity of the proteins that were differentially cisplatin cross-linked in the prostate benign and cancer cell lines was determined by mass spectrometry (in-gel digestion, nanoliquid chromatography and tandem mass spectrometry). Spot 2 was found in prostate cancer preparations, but much less so in the benign BPH-1 cell preparation (Fig. 1a). Spot 2 was identified as KH-type splicing regulatory protein (KSRP) or far upstream element-binding protein 2 (FUBP2) encoded by the *KHSRP* gene (Fig. 1c). KSRP/FUBP2 has a molecular mass of 73.1 kDa and pI of 6.85.

Spot 16 was among the proteins cisplatin cross-linked to DNA in DU 145 cells, but was absent in BPH-1, LNCaP and PC-3 cells. This protein was identified as UCHL1 which has a molecular mass of 24.8 kDa and a pI of 5.33 (Fig. 1d).

To verify the identification of proteins in spots 2 and 16 as KSRP/FUBP2 and UCHL1, respectively, immunoblot analyses using anti-KSRP or anti-UCHL1 antibodies were performed on samples of proteins isolated by cross-linking to DNA with cisplatin in human prostate benign and cancer cell lines. HDAC1, which we had previously shown to be cross-linked to DNA by cisplatin or formaldehyde [17], was used as positive control and was also detected by immunoblot. KSRP/FUBP2 was cross-linked to DNA in the prostate cancer cell lines, but not in BPH-1 (Fig. 1c). In contrast, UCHL1 was cross-linked to DNA with cisplatin in DU 145, but not BPH-1, PC-3 and LNCaP cells (Fig. 1d). To determine whether UCHL1 could be cross-linked to nuclear DNA with formaldehyde under ChIP assay conditions, cells were treated with formaldehyde, and proteins cross-linked to DNA were collected after dissociation from hydroxyapatite-bound DNA. As with cisplatin cross-linking, UCHL1 was cross-linked to nuclear DNA only in the DU 145 cells (Fig. 1d).

The differential UCHL1 cross-linking among the four cell lines could be due to differences in recruitment or differences in expression of UCHL1. Thus, we determined the cellular expression of UCHL1 in the prostate benign and cancer cell lines. Figure 1d shows that UCHL1 was expressed only in DU 145 cells. Moreover, UCHL1 was not expressed in the LNCaP castration-resistant cell line C4-2 (data not shown) [18]. *UCHL1* transcripts were found in DU 145, but not in PC-3 or C4-2 cells (data not shown).

We also tested a panel of PC-3-derived cell lines. The PC3M, PC3-Pro4 and PC3-LN4 cell lines were obtained by injection into the prostate of athymic mice, isolation from prostate and lymph nodes and re-injection into the prostate. These PC-3 lines differ in metastatic potential, with the PC3-LN4 having the greatest metastatic potential [19]. We found that, contrary to the parental PC-3 cell line, these three cell lines expressed UCHL1 (Fig. 1e).

We investigated whether UCHL1-expressing cells in general had UCHL1 associated with nuclear DNA or whether this was a feature unique to DU 145 cells. In all cell lines in which UCHL1 was expressed (PC3M, PC3-Pro4 and PC3-LN4), UCHL1 was cross-linked to nuclear DNA by formaldehyde (Fig. 1e). HEK293T cells, which have characteristics of immature neurons [20], were reported to express UCHL1 [16]. Our immunoblot analysis confirmed this finding, but showed that UCHL1 levels were lower in HEK293T than in DU 145 cells (Fig. 1e). In agreement with the above results, UCHL1 was cross-linked to DNA in HEK293T cells. However, we consistently observed that the yield of UCHL1 recovered from formaldehyde-treated HEK293T cells was lower than that from DU 145 cells.

These results demonstrate that in UCHL1-expressing cells, UCHL1 is associated with nuclear DNA.

### Genomic distribution of UCHL1

To determine the genomic location of UCHL1 in DU 145 cells, we initially performed chromatin immunoprecipitation (ChIP) sequencing (ChIP-seq) with formaldehyde cross-linking and sonication to fragment the DNA (average lengths 200–300 bp). We tested several commercial UCHL1 antibodies for specificity and efficiency in immunoprecipitating UCHL1 under ChIP conditions. The mouse monoclonal anti-UCHL1 antibody (R&D Systems) was identified to be “CHIP-grade.” ChIP-seq and bioinformatic data analyses revealed that the DNA sequences of the UCHL1 peaks were highly repetitive. These sequences contained a CCC (GGG) repeat every three bases. The UCHL1 ChIP-sequencing was repeated in DU 145 and HEK293T cells with two revisions to the method. First, we used dual cross-linking (DSP and formaldehyde), which we found increased the association of UCHL1 to DNA. Secondly, we fragmented the chromatin with micrococcal nuclease. Significantly, read-enriched positions or peaks were recognized using the MACS software default parameters with a *p* value threshold of 1E−5 and mfold (30,10). We identified 191 peaks in the two DU 145 UCHL1 ChIP-seq analyses (Additional file 1).

**Fig. 2:**
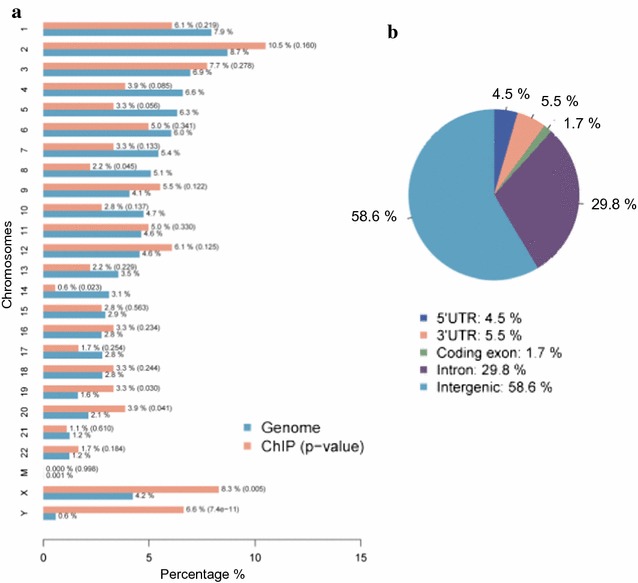
Genomic distribution of UCHL1 in DU 145 cells. **a** Chromosome distribution of UCHL1 binding sites: Blue bars represent the relative lengths of each chromosome, and red bars represent the percentage of UCHL1 peaks (out of 191 peaks) per chromosome. *P* value indicates the significance of the enrichment of ChIPped regions relative to the genome background and is shown in parentheses. **b** UCHL1 binding sites were divided according to their relative locations to gene loci

Analyses of the 191 UCHL1 ChIP-seq peaks in DU 145 showed that although UCHL1 binding sites were located on all chromosomes, the UCHL1 peaks were enriched on chromosomes 2, 12, 20, X and Y (Fig. 2a). Most UCHL1 peaks were present in intergenic regions (about 60%) and introns (about 30%) (Fig. 2b). Few UCHL1 peaks were found in exons (about 2%). We identified 76 genes for which the UCHL1 peaks were located within the gene body or 5-Kb up or downstream of the gene (Additional file 2). The functional GO term enrichment of these 76 genes showed several genes with roles in Rho signaling (Additional file 3).

**Fig. 3:**
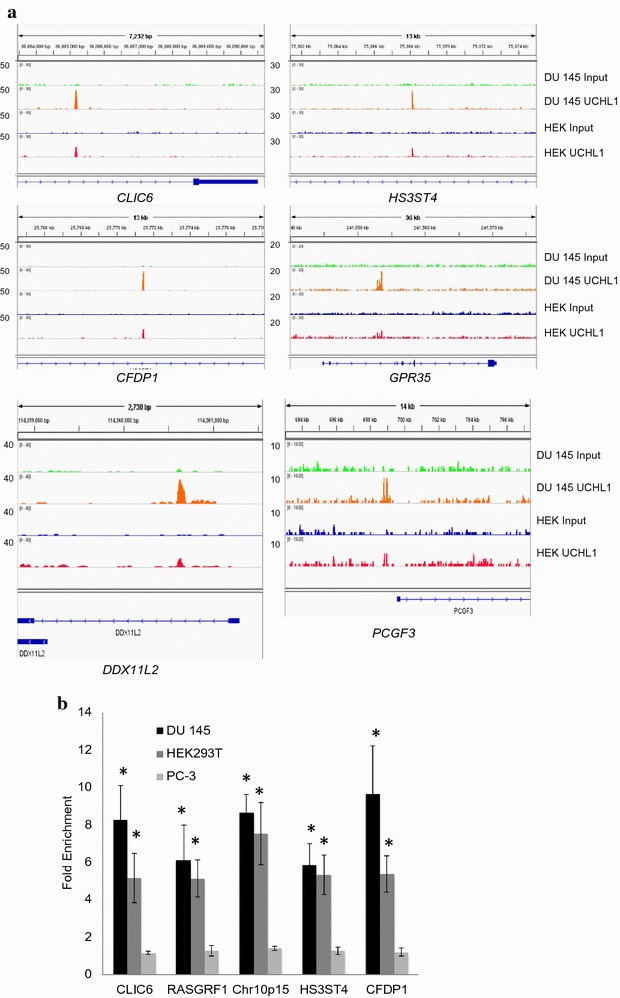
Representation and validation of random UCHL1 binding sites in DU 145 and HEK293T cells. **a** Signal tracks showing DNA enrichment in UCHL1 ChIP compared to genomic input. The peak signals were normalized by igvtools. **b** ChIP experiments were performed using anti-UCHL1 antibodies on DSP/formaldehyde cross-linked 300–400-bp chromatin fragments obtained by sonication from DU 145, HEK293T or PC-3 cells. Fold enrichment values were normalized to IgG ChIP values. Asterisk (*) indicate statistical significance *p* < 0.05

Of the 191 UCHL1 peaks in DU 145 cells, 55 were also detected in HEK293T cells (Additional file 1). In HEK293T cells, the intensity of UCHL1 peaks was lower than in DU 145 cells, consistent with the lower level formaldehyde cross-linking of UCHL1 to HEK293T nuclear DNA (Fig. 1). UCHL1 peaks were identified in the introns of genes coding for *CLIC6* (chloride intracellular channel 6), *CFDP1* (craniofacial development protein 1), *HS3ST4* (heparan sulfate-glucosamine 3-sulfotransferase 4), *GPR35* (G protein-coupled receptor 35), *DDX11L2* (DEAD/H-box helicase 11 like 2) and the upstream promoter region of the *PCGF3* (polycomb group ring finger 3) gene (Fig. 3a). Dual cross-linking (1 mM DSP + 0.5% formaldehyde) assays were applied to validate the UCHL1 binding sites in DU 145 and HEK293T cells. As a control, we also performed the UCHL1 ChIP assays with PC-3 prostate cancer cells which do not express UCHL1. Figure 3b shows the association of UCHL1 to the introns of *CLIC6*, *RASGRF1*, *HS3ST4*, *CFDP1* and to the intergenic site at chr10: 3,986,143–3,986,281 in DU 145 and HEK293T cells.

**Fig. 4:**
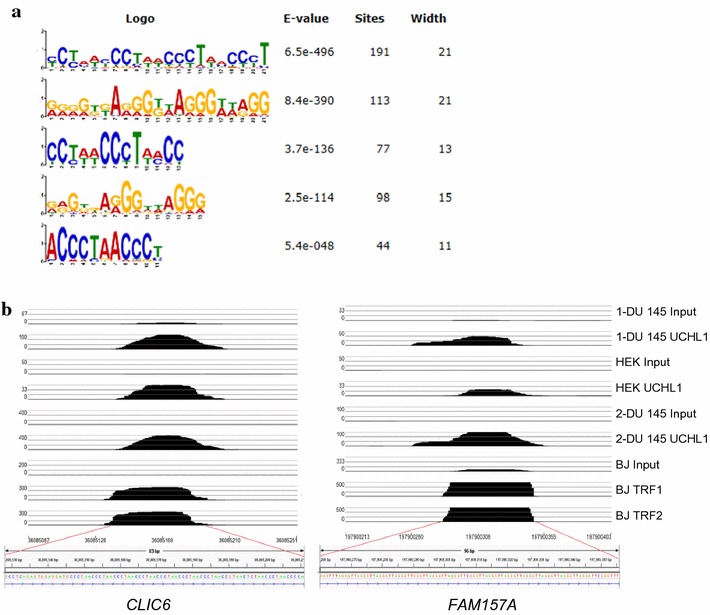
UCHL1 localizes to telomeric repeat sequences as do TRF1 and TRF2. **a** The top five motifs were identified using the MEME software. The *E* value is the expected number of hits by chance (the lower the *E* value the more significant is the motif). **b** Signal tracks showing DNA enrichment in UCHL1 ChIP from DU 145 and HEK293T cells and TRF1 or TRF2 ChIP from BJHELTRas^mc^ cells (Simonet et al. [21]—21423270) were aligned for *CLIC6* and *FAM157A* genes. Cells were treated with formaldehyde (1-DU 145 Input and UCHL1, HEK Input and UCHL1) or DSP/formaldehyde (2-DU 145 Input and UCHL1)

We extracted the sequences of the 191 common peaks for motif analysis, using the MEME program. We found five top significant motifs (Fig. 4a). The UCHL1 peaks often located with the simple repeat (CCCTAA)*n*.

### UCHL1 is associated with the shelterin complex

As there is no evidence that UCHL1 binds DNA directly, we surmised that UCHL1 was associating with a DNA-binding protein that was recognizing the UCHL1 motif. The sequences of UCHL1 binding sites on genes *DDX11L2* and *PCGF3* were extracted and searched against PROMO (TRANSFC database version 8.3). We found that the TRF1 (telomeric repeat binding factor 1) binds to the sequence underlying the UCHL1 peaks. TRF1 is part of the shelterin complex along with TRF2, TIN2, TPP1, POT1 and RAP1 (telomeric repeat binding factor 2-interacting protein 1, also known as TERF2IP).

Using the publicly available TRF1 and TRF2 ChIP-seq data (GEO Series accession number GSE26005) [21], we show in Fig. 4b that several DNA regions associated with the UCHL1 binding sites in DU 145 and HEK293T cells aligned with genomic locations of TRF1 and TRF2 peaks in the BJHELTRas^mc^ tumor cell line. BJHELTRas^mc^ are human fibroblasts (BJ) cells rendered tumorigenic by successive retroviral transductions of telomerase, simian virus 40 early region and oncogenic *RAS* [22]. The 191 UCHL1 common peaks in DU145 share 28 TRF1 peaks, 26 TRF2 peaks, respectively, or 24 TRF1/TRF2 peaks. This provided evidence that UCHL1 localizes to the same genomic locations as the shelterin complex and the underlying sequences of these peaks contained the canonical telomeric repeat sequences, CCCTAA (TTAGGG) found at telomeres and interstitial telomeric sites (ITSs). UCHL1 binding sites containing this repeat are summarized in Table 1.

**Table 1:** List of UCHL1 binding sites containing CCCTAA (TTAGGG) repeats within and 5-kb up or downstream genes in DU 145 cells

Chr	Peak location			Expression level^b^
	Start	End	Gene name	
chr1	10,102	10,425	Intergenic, upstream of DDX11L1^a^	–
chr1	18,580,820	18,580,945	Intron (IGSF21, intron 2 of 9)	–
chr2	75,299,579	75,299,730	Intron (TACR1, intron 2 of 4)	–
chr2	114,360,577	114,360,716	Intron (DDX11L2, intron 1 of 2)^a^	–
chr2	182,140,505	182,140,611	Intron (LOC101927156, intron 3 of 4)	NA
chr2	241,553,483	241,553,609	Intron (GPR35, intron 2 of 5)^a^	–
chr3	9,736,179	9,736,298	Intron (MTMR14, intron 17 of 17)	++
chr3	159,411,986	159,412,107	Intron (SCHIP1, intron 1 of 6)	++/−
chr3	197,900,087	197,900,374	Intron (FAM157A, intron 6 of 6)^a^	–
chr3	197,901,288	197,901,414	Intron (FAM157A, intron 6 of 6)^a^	–
chr4	698,901	699,003	Promoter–TSS (PCGF3)	++
chr5	82,811,907	82,812,020	Intron (VCAN, intron 6 of 12)^a^	–
chr6	147,601	147,760	Intron (LINC00266-3, intron 1 of 2	NA
chr6	38,912,686	38,912,816	Intron (DNAH8, intron 79 of 92)	–
chr6	161,866,788	161,866,892	Intron (PARK2, intron 8 of 10)	–
chr9	10,007	10,127	Intergenic, upstream of DDX11L5^a^	NA
chr9	10,153	10,269	Intergenic, upstream of DDX11L5^a^	NA
chr9	10,288	10,403	Intergenic, upstream of DDX11L5^a^	NA
chr9	2,824,012	2,824,113	Intron (KIAA0020, intron 11 of 17)	++
chr9	141,054,226	141,054,361	Intron (TUBBP5, intron 1 of 4)^a^	+
chr10	4,111,307	4,111,412	Intron (LOC101927964, intron 1 of 3)	NA
chr12	22,393,946	22,394,047	Intron (ST8SIA1, intron 3 of 3)	–
chr15	42,243,142	42,243,265	Intron (EHD4, intron 2 of 5)	+++
chr16	59,970	60,077	Intergenic, upstream of DDX11L10	–
chr16	25,771,380	25,771,526	Intron (HS3ST4, intron 1 of 1)^a^	–
chr16	75,368,040	75,368,166	Intron (CFDP1, intron 5 of 6)	++
chr16	88,946,595	88,946,716	Intron (CBFA2T3, intron 9 of 10)	–
chr18	10,273	10,379	Intergenic, upstream of LOC102723376^a^	NA
chr20	10,172,151	10,172,266	Intron (SNAP25-AS1, intron 2 of 4)^a^	NA
chr20	62,918,067	62,918,182	Intergenic, upstream of LINC00266-1^a^	–
chr20	62,918,353	62,918,469	Intergenic, upstream of LINC00266-1^a^	–
chr20	62,918,583	62,918,739	Intergenic, upstream of LINC00266-1^a^	–
chr21	36,085,122	36,085,247	Intron (CLIC6, intron 5 of 5)	–
chr21	45,851,799	45,851,900	Intron (TRPM2, intron 27 of 31)	+
chr22	45,022,364	45,022,483	Intergenic, upstream of LINC00229^a^	–

^a^Identifies binding sites also detected in HEK293T cells

^b^Expression levels in DU 145 cells were extracted from NCBI GEO GSE71756 and GSE71208

**Fig. 5:**
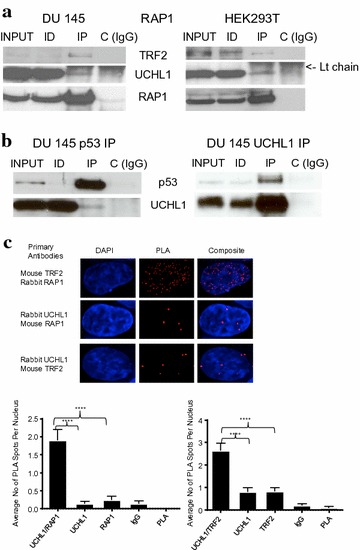
UCHL1 interacts with the shelterin complex and the nuclear scaffold. **a** DU 145 or HEK293T cellular lysates in a low stringency buffer were incubated with anti-TRF2 antibodies or control IgG. The immunoprecipitate (IP), and equal volumes of lysate (Input) and immunodepleted (ID) fractions were analyzed by immunoblotting with the indicated antibodies. **b** In left panel, DU 145 cellular lysate in a low stringency buffer was incubated with anti-p53 antibodies or control IgG. In right panel, cell lysate in a high stringency buffer of DU 145 cells treated with the cross-linker DSP was incubated with anti-UCHL1 antibodies or control IgG. The immunoblot analysis was done as in (**a**). **c** In situ PLA images of the interaction between TRF2 and RAP1 (positive control), UCHL1 and RAP1 or UCHL1 and TRF2 in HEK293T cells. PLA signals appear as discrete red dots and nuclei are visualized by DAPI (blue). A total of 30 nuclei per group were quantified. The average number of PLA foci per nucleus was graphed with error bars representing standard errors of the means. Single primary antibodies, isotype control and PLA probes only were used as negative controls as indicated. ****p < 0.0001 (determined by one-way ANOVA)

Using a strategy to identify low affinity or transient interactions of proteins with the shelterin complex, UCHL1 was shown to associate with RAP1 in HTC75 cells [23]. In co-immunoprecipitation assays, we observed an interaction, albeit weak, between TRF2 and UCHL1 in DU 145 and HEK293T cells (Fig. 5a). The interaction between TRF2 and RAP1, used as positive control, was much stronger. We attempted to reverse the order by immunoprecipitating UCHL1 first followed by immunoblotting with RAP1 or TRF2. However, this approach was unsuccessful in showing an interaction between UCHL1 and RAP1 or UCHL1 and TRF2. As a control, we determined the interaction between UCHL1 and p53, which has been demonstrated previously [9, 24]. In DU 145 cells, we confirmed that p53 binds to UCHL1 (Fig. 5b).

We next applied the proximity ligation assay (PLA) to observe interactions between UCHL1 and the shelterin proteins RAP1 and TRF2 in HEK293T cells. The TRF2–RAP1 interaction was used as a positive control [25] (Fig. 5c). Appropriate single primary antibodies, isotype control and PLA probes only were used as negative controls (Additional file 4). A total of 30 nuclei per group were quantified. Interactions were observed between UCHL1 and RAP1 and between UCHL1 and TRF2. However, the number of interactions was considerably less than that observed between RAP1 and TRF2 (Fig. 5c).

**Fig. 6:**
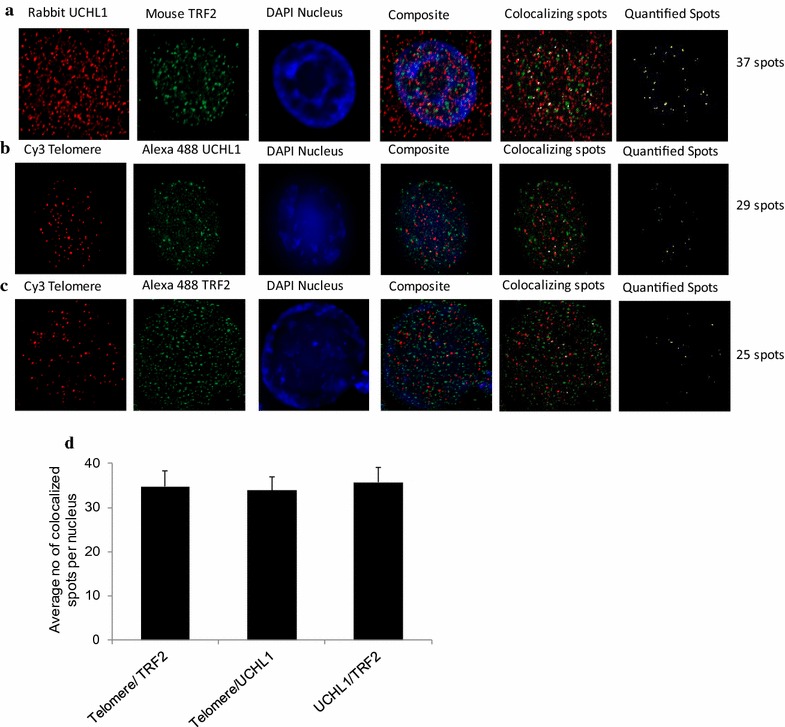
UCHL1 interacts with TRF2 and co-localizes with telomeres. **a** Double immunofluorescence displayed co-localization of TRF2 and UCHL1 in HEK293T cells. Blue—nucleus; red—UCHL1; green—TRF2; yellow—TRF2–UCHL1 co-localizing spots. **b** Telomeric localization of UCHL1 was determined in HEK293T cells by immuno-FISH with antibodies against UCHL1 and fluorochrome-coupled (Cy3) telomere PNA probes. **c** Telomeric localization of TRF2 was determined in HEK293T cells by immuno-FISH for antibodies against TRF2 and fluorochrome-coupled (Cy3) telomere PNA probes. Blue—nucleus; red—telomeres; green—UCHL1/TRF2; yellow—telomere-UCHL1/TRF2 co-localizing spots. **d** Thirty HEK cell nuclei per experiment were quantified. Average number of colocalized spots per nucleus was counted and graphed. Image J software was used for quantification. Error bar represents the standard error of mean

Double immunofluorescence experiments with antibodies against UCHL1 and TRF2 were done to determine whether we could observe co-localization of these two proteins in HEK293T cells. A low level of co-localization of UCHL1 and TRF2 was observed, with most UCHL1 and TRF2 having distinct locations (Fig. 6a). In immuno-FISH studies, we determined the co-association of UCHL1 or TRF2 with the telomere. For both proteins, there was a low level of association with the telomere (Fig. 6).

Together, these observations are consistent with a weak or transient interaction between UCHL1 and the shelterin proteins.

### Mutant p53 is not associated with UCHL1-binding sites

The (TTAGGG)*n* repeat has the potential to form a G-quadruplex structure. Several loci that UCHL1 interacts with (chr 18 subtelomeric region, upstream promoter region of *PCGF3* and intron of *DDX11L2*) have the potential to form G-quadruplexes in DU 145 cells [26]. TRF2, POT1 and mutant p53 are G-quadruplex-binding proteins [27, 28]. As HEK293T and DU 145 cells express mutant p53 [29], it was conceivable that mutant p53 was recruiting UCHL1 to the ITS and telomeres. We performed p53 ChIP assays with dual crossed-linked PC-3, HEK293T and DU 145 cells. The PC-3 cells do not express p53 and thus served as a negative control in the ChIP assays (Additional file 5a). We did not observe an association with the five UCHL1 binding sites tested (Additional file 5b). We conclude that mutant p53 is not involved in recruiting UCHL1 to the ITS.

### UCHL1 and RAP1 are associated with the nuclear scaffold

**Fig. 7:**
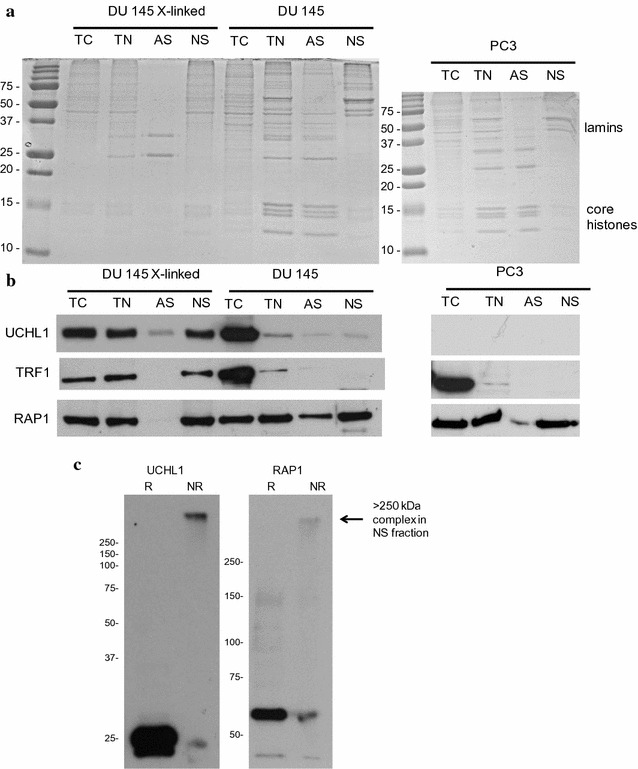
UCHL1 and RAP1 are associated with the nuclear scaffold. **a** Proteins (5 µg) from each fraction [total cellular protein (TC), total nuclear protein (TN), ammonium sulfate fraction (AS) and nuclear scaffold protein (NS)] were resolved on a SDS 15% polyacrylamide gel and stained with Coomassie Blue. The DU 145 cells were treated or not with protein–protein cross-linker DSP. **b** Immunoblot analysis using the indicated antibodies on TC, TN, AS and NS proteins from DU 145 cells treated or not with protein–protein cross-linker DSP. PC-3 cells were not treated with DSP. **c** Immunoblot analysis of UCHL1 and RAP1 in nuclear scaffold protein complexes from DSP-treated DU 145 cells. The nuclear scaffold was solubilized in SDS sample buffer in both reducing (R) and non-reducing (NR) conditions and analyzed by SDS-PAGE on a 10% gels and immunoblotting

The nuclear scaffold, also referred to as nuclear matrix, is formed by a network of protein–RNA interactions. It has a central role in organization of the chromatin structure [30] and provides a platform for nuclear processing such as transcription, replication and RNA processing [31–39]. It had been reported that TIN2 was associated with the nuclear scaffold in normal human fibroblasts [40]. We initially observed that UCHL1 was associated with DU 145 nuclear DNA by using the cross-linker cisplatin. As cisplatin preferentially cross-links nuclear scaffold proteins to DNA [41], we determined whether UCHL1 was a nuclear scaffold associated protein. DU 145 nuclei were digested extensively with DNase I, and chromatin was extracted with 0.25 M ammonium sulfate. The nuclear scaffold fraction was depleted in histones and enriched in lamins (Fig. 7a). Figure 7b shows that RAP1 and, to a lesser extent, UCHL1 were associated with the nuclear scaffold of DU 145 cells. RAP1 was also associated with the nuclear scaffold of PC-3 cells (Fig. 7b). We next tested whether the protein–protein cross-linker DSP would increase the retention of UCHL1 and TRF1 to the nuclear scaffold of DU 145 cells. With the DSP-treated cells, there was a marked increase in UCHL1 and TRF1 with the nuclear scaffold (Fig. 7b). Further, there was a decrease in the amount of RAP1 in the ammonium sulfate fraction. These observations suggest that RAP1 may have a role in recruiting the shelterin complex and UCHL1 to the nuclear scaffold.

To determine whether UCHL1 was associated with the nuclear scaffold bound RAP1, DU 145 cells were cross-linked with DSP before isolating the nuclear scaffold. The nuclear scaffold was solubilized in SDS sample buffer, and the proteins as cross-linked complexes were electrophoretically resolved on a 10% polyacrylamide SDS gel and analyzed by immunoblotting with anti-UCHL1 antibodies. In non-reducing conditions, the UCHL1 and RAP1 barely resolved in the gel and were in a very large complex with a molecular mass in excess of 250 kDa (Fig. 7c). In reducing conditions which break the cross-links, UCHL1 and RAP1 were observed. To determine whether UCHL1 and RAP1 were associated in the large complex, we attempted co-immunoprecipitation experiments in which UCHL1 was immunoprecipitated from the solubilized nuclear scaffold protein fraction from DSP cross-linked DU145 cells. Unfortunately, we could not generate a conclusive result since UCHL1 and RAP1 both electrophoresed at the same position as the light and heavy IgG chains (Additional file 6). However, we did not observe either a UCHL1 or RAP1 band in the immunodepleted (ID) fraction, suggesting that both UCHL1 and RAP1 were together in the large protein complex.

### Knockdown of UCHL1 does not alter RAP1 and TRF2 levels

**Fig. 8:**
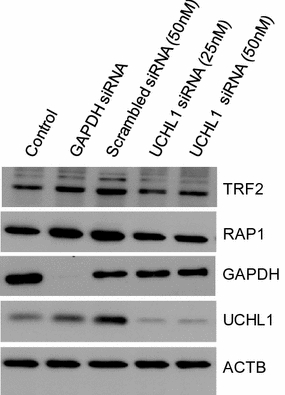
UCHL1 knockdown and shelterin protein levels. HEK293T cells were transfected with GAPDH siRNA (50 nM), scrambled siRNA control (50 nM) and two UCHL1 siRNA with the final concentrations of 25 and 50 nM. Whole protein lysates were extracted from control and siRNA-transfected HEK293T cells 72 h posttransfection, and immunoblot analysis was performed with antibodies against UCHL1, RAP1, TRF2 and GAPDH. Beta actin was used as an internal control

As UCHL1 protects proteins (NOXA, p53, HIF-1-alpha, EGFR) from turnover by removing polyubiquitin chains [6–10], it was possible that UCHL1 had a role in regulating the turnover the shelterin proteins. To test this idea, we performed UCHL1 knockdown experiments with HEK293T cells. In the cells in which there was a reduction of 93% in UCHL1 levels, there was a minimal effect on the levels RAP1 (6% reduction) and TRF2 (Fig. 8). We conclude that UCHL1 does not have a role in the stabilization of RAP1.

## Discussion

UCHL1 has multiple roles in ubiquitin homeostasis, protein stability and tumor progression. In this study, we discovered a new role for UCHL1 in associating with nuclear DNA. In addition to UCHL1-expressing prostate cancer cells and HEK293T cells, we are in the process of characterizing other cell types including lung cancer and medulloblastoma cell lines. We are finding that when UCHL1 is expressed, the protein is associated with DNA (unpublished data).

Cisplatin or formaldehyde cross-linking in combination with hydroxyapatite chromatography has been a productive approach to discover proteins that are differentially associated with nuclear DNA in cells. Our finding that KSRP/FUBP2 was cross-linked to DNA in prostate cancer cell lines supports the validity of our approach as KSRP is a known single-stranded DNA-binding protein upregulated in some cancers [42, 43]. Previously, we used this method to demonstrate that peroxiredoxin 1 (PRDX1) was associated with the nuclear DNA in triple negative breast cancer cells [17]. As with PRDX1, UCHL1 binds DNA indirectly through interaction with a DNA-binding protein, which for UCHL1 includes the shelterin complex. UCHL1 and the shelterin complex bind at telomeres and interstitial TTAGGG repeats located throughout the mammalian cell genome. There is mounting evidence that ITS plays a role in genomic instability and in human disease [44–47].

Although tandem (TTAGGG)*n* repeats are found primarily at telomeres, approximately 40% of the (TTAGGG)*n* sequences locate to pericentric and ITSs [48]. There are three classes of ITSs in the human genome (short, long subtelomeric and fusion) [49]. The short ITSs are found at over 50 loci in the human chromosome. In contrast to telomeres which stabilize chromosomes, ITSs destabilize chromosomes [50] and some ITSs coexist with fragile sites [49]. Site-specific insertion of a 800-bp (TTAGGG)*n* sequence into the intron of the Chinese hamster *APRT* gene resulted in a 30-fold increase in gene rearrangements with the (TTAGGG)*n* repeat [50]. UCHL1 is located with ITSs in the regions 2q31 and 21q22, which demonstrate instability, and at ITSs in 2q14 which is an aphidicolin-inducible common fragile site [51, 52].

The insertion of the (TTAGGG)*n* repeat into the intron did not affect the expression of the *APRT* gene. In DU 145 prostate cancer cells, UCHL1 was frequently found at ITSs located in the intron of genes that were either expressed or silent. Several reports show that TRF2 regulates gene expression through binding to ITSs located within the upstream promoter region or in introns, and UCHL1 may modify this TRF2 activity [22, 53].

It is possible that UCHL1 has a role in regulating gene expression through changes in the stabilization of shelterin proteins. As several shelterin proteins (TRF1, TRF2, POT1 and RAP1) are ubiquitinated, UCHL1 may have a role in regulating the stability of these proteins. However, our studies do not provide support for such a function. UCHL1 may regulate the nuclear location of shelterin-associated genomic regions by regulating the association of DNA sequences with the nuclear scaffold. It had been reported that TIN2 is bound to the nuclear scaffold [40]. We show here that RAP1 is associated with the nuclear scaffold in DU 145 cells and PC-3 cells, which lack UCHL1 expression. This observation suggests that RAP1 is key in recruiting the shelterin complex to the nuclear scaffold along with the shelterin-bound ITS. Whether UCHL1 has a role in modulating the interaction between shelterin-bound telomere/ITS, lamins and the nuclear scaffold remains to be determined [54, 55].

## Conclusions

Deregulated expression and mutations in UCHL1 are associated with human diseases including cancer, Parkinson’s disease, Alzheimer disease and type 2 diabetes [56–61]. The newly discovered ability of UCHL1 to associate with components of the shelterin complex and telomeric/ITSs may be relevant in these disease processes.

## Methods

### Cell culture

BPH-1, LNCaP, PC-3, DU 145 and HEK293T cell lines were obtained from the American Type Culture Collection. PC-3M, PC-3M LN4 and PC-3M Pro cells were obtained from Dr. Sui Huang and C4-2 cells from Dr. Paul Rennie. BPH-1, LNCaP, PC-3, PC-3 derived and C4-2 cells were grown in RPMI-1640 medium (Life Technologies). DU 145 cells were grown in EMEM medium (Life Technologies) and HEK293T cells in DMEM medium (Life Technologies). All the media were supplemented with 10% FBS and 1% antibiotic–antimycotic (Life Technologies). The cells were grown at 37 °C with 5% CO_2_. DU 145 and HEK293T cell lines were authenticated by STR DNA profiling analysis [62].

### Isolation and analysis of proteins cross-linked to DNA

Cells were incubated with 1 mM cisplatin at 37 °C for 2 h or with 1% formaldehyde at room temperature for 10 min, and proteins cross-linked to DNA in situ were isolated and resolved by two-dimensional gel electrophoresis. The methods to isolate and identify proteins differentially associated with DNA were described previously [17].

### DSP cross-linking of proteins, immunoprecipitation (IP) and immunoblotting

These procedures were performed as described [63]. To cross-link proteins, cells were incubated with 1 mM DSP (dithiobis[succinimidylpropionate]) (Thermo Fisher Scientific) at room temperature for 30 min. Antibodies against UCHL1 (R&D Systems, Inc.), Lamin A/C (Upstate Biotechnology), HDAC1 (Millipore), actin (Sigma-Aldrich), GAPDH (Santa Cruz Biotechnology), p53 (Abcam), TRF1 (Novus Biologicals), TRF2 (Novus Biologicals), RAP1 (TERF2IP) (Bethyl Laboratories) and KSRP (a gift from Dr. Douglas L. Black) were used.

### ChIP and ChIP-seq assays

ChIP experiments were done as previously described [64] with the following modifications. In one protocol, DU 145 cells were treated with 1% formaldehyde for 10 min at room temperature and chromatin was submitted to sonication to generate fragments of an average length of 200–300 bp. In a second protocol, DU 145 and HEK293T cells were incubated with 1 mM DSP for 30 min at room temperature and then with 0.5% formaldehyde for 10 min. Dual cross-linked chromatin was processed to mononucleosomes (> 60% mononucleosomes) by micrococcal nuclease (Worthington Biochemical Corporation). The mouse monoclonal anti-UCHL1 antibody (R&D Systems) was used to immunoprecipitated chromatin fragments. Primers used to amplify ChIPped DNA were as follows: CLIC6 forward, 5′-CTAACCCTAACCGTAACTCTAAC-3′, and reverse, 5′-CTCCCAAGCACCTTGTC-3′; RASGRF1 forward, 5′-CCTTCCCTATCCTCCCTTAT-3′, and reverse, 5′-CAAGTTCCCTTCTCACTCTG-3′; Chr10p15 forward, 5′-CCACTGCTGAGTTACAGGCA-3′, and reverse, 5′-CCCTGGTCAAAATGTCATCC-3′; HS3ST4 forward, 5′-GTGCGTCTATGAGGGCTGCCA-3′, and reverse, 5′-AGGCACCCAGTGGCTTTAACAGT-3′; CFDP1 forward, 5′-TTGCCAGCCCCACCCACTCT-3′, and reverse, 5′-TCCCGGTCCTTTTTACCCGTCTGT-3′. Enrichment values are the mean of three independent experiments. Error bars indicate standard error.

### ChIP DNA preparation for SOLiD ChIP-sequencing

The ChIP and input DNA for ChIP-seq were purified by magnetic AgencourtAMPure XP beads (Beckman Coulter) as per manufacturer’s instructions. The DNA was quantified by the Qubit 2.0 Fluorometer (Life Technologies). The subsequent ChIP-seq library preparation steps were performed according to the 5500 SOLiD fragment library protocol (Life technologies). The ChIP and input DNA size was first analyzed by the high-sensitivity DNA kit and 2100 Bioanalyzer (Agilent Technologies). Approximately 20 ng of ChIP DNA was end-repaired and size-selected (100–250 bp). This was followed by dA tailing and ligation of SOLiD barcodes. Finally, DNA libraries were prepared by ePCR and loaded onto the flowchip for sequencing by the SOLiD platform.

### SOLiD sequence mapping

A total of 181,900,271 and 70,075,439 50-base SOLiD UCHL1 ChIP-seq sequence reads were generated from four DU 145 and two HEK293T cell samples, respectively. We also analyzed three samples of 19,524,349 sequence reads of the SOLiD ChIP-seq data from NCBI GEO (GSE26005). All these sequences were ensured by quality check (noise to signal ratio and bamStats after mapping). The sequence reads were mapped on the human reference genome (hg19) using Lifescope v2.5.1 software (Life Technologies) with 2-mismatch settings. The GSE26005 sequences were mapped using Bowtie version 2 [65].

### Peak calling and annotation

The binding site candidates were identified by comparing ChIP sample to input sample using the model-based analysis of ChIP-seq (MACS) peak caller [66] with a *p* value 1e−5 and an mfold cutoff of high limit of 30 and low limit of 10. We used the software CEAS [67] to analyze the peak distribution among the genome and their location regarding to genes. We used the Ensembl 75 for gene annotations. We searched the binding site sequences against the TRANSFAC Professional v9.3 database (http://www.biobase.de) by Match program with minimal false discovery rate of 0.1 cutoff. The common peaks among different samples were analyzed by custom script. We determined that two peaks overlapped if one peak’s center fell into another peak’s range.

### Data viewing

Browser views of gene tracks, ChIP-seq data and peaks are shown using Integrated Genomics Viewer (IGV) after data normalization using igvtools [68] or Partek Genomics Suite v6.6.

### Motif analysis

The sequences of the 191 peaks were extracted from the human genome hg19 reference using the peak bed file and bedtools. The motifs among these sequences were analyzed by MEME discovery tools [69] with W:5–25 settings.

### GO term analysis

The 76 genes corresponding to 191 UCHL1 peaks were obtained using software HOMER [70]. Further, these 76 genes were subjected to GO analysis using software DAVID [71].

### Double immunofluorescence

HEK293T cells were seeded and cultured overnight on 3-aminopropyl triethoxysilane (APTES, Sigma) coated glass slides. The cells were fixed for 20 min in 3.7% formaldehyde (Fisher Scientific), permeabilized using 0.25% Triton X-100 and blocked with 4 × SSC/4%BSA for 1 h at room temperature. Rabbit antibodies against PGP9.5 (UCHL1) (Abcam) and mouse antibodies against TRF2 (Novus Biologicals) were incubated for 1 h and then for 1 h again at room temperature with secondary antibodies Alexa Fluor (AF) 488 conjugated goat anti-rabbit IgG and AF594 conjugated goat anti-mouse IgG, respectively (both Life technologies). Slides were then counterstained with DAPI (Sigma), mounted with Fluoromount G (Southern Biotech) and imaged with a Zeiss Z1 microscope using a 63x oil immersion objective with NA of 1.4 and image J software.

### Immuno-FISH

HEK293T cells were grown overnight on APTES-coated glass slides, fixed with 3.7% formaldehyde in PBS for 20 min at RT and permeabilized with 0.25% Triton X-100 for 10 min at room temperature. After blocking for 1 h with 4% BSA in 4x SSC buffer, slides were incubated for 1 h at room temperature with primary antibodies to UCHL1 (rabbit monoclonal, Abcam) and TRF2 (mouse monoclonal, Novus Biologicals). After washing, pepsin (Sigma) digestion was done for 4 min at 37 °C. Cells were postfixed in 3.7% formaldehyde for 2 min at room temperature and submitted to dehydration through ethanol series. Fluorochrome-coupled (Cy3) Telomere PNA probe (DAKO) was applied (5 μl probe/slide), and following denaturation at 80 °C for 3 min, hybridization was done for 2 h at 30 °C. Slides were washed in 70% deionized formamide (Sigma) in 10 mM Tris pH 7.4, 2 × SSC (5 min at 55 °C), 0.1 × SSC and 2 × SSC/0.05%Tween-20 at room temperature. Secondary antibody AF488 goat anti-rabbit IgG or AF488 goat anti-mouse IgG was applied to the slides and incubated for 1 h at RT. Slides were washed in 2 × SSC/0.05% Tween 20, counterstained with DAPI (Sigma), mounted with Fluoromount G (Southern Biotech) and imaged with a Zeiss Z1 microscope using a 63 × oil immersion objective with NA of 1.4 and Zen Software (Zeiss).

### Proximity ligation assay

PLA experiments were done on HEK293T cells, using the Duolink kit (Sigma-Aldrich) as previously described [25]. Antibodies used were anti-UCHL1 rabbit monoclonal (Abcam), anti-TRF2 mouse monoclonal (Novus Biologicals), anti-RAP1 (TERF2IP) mouse monoclonal (Abcam), anti-RAP1 (TERF2IP) rabbit polyclonal (Bethyl Laboratories), rabbit IgG and mouse IgG (Sigma-Aldrich).

### Nuclear scaffold protein isolation and immunoprecipitation

Nuclear scaffold proteins were isolated from DU 145 cells as described previously [72]. Cross-linking was performed at room temperature for 30 min with 1 mM DSP (dithiobis[succinimidylpropionate]) (Thermo Fisher Scientific). Then, cells submitted or not to cross-linking were resuspended in TNM buffer (100 mM NaCl, 300 mM sucrose, 10 mM Tris–HCl, pH 7.4, 2 mM MgCl_2_ and 1% thiodiglycol v/v) containing 1 mM PMSF, 30 mM sodium butyrate and phosphatase inhibitors. Triton X-100 was added to a final concentration of 0.25% (v/v). An aliquot of this supernatant was saved as the total cellular protein fraction. Nuclei (20 A_260_/ml) were digested with 100 µg/ml DNase 1 (Sigma-Aldrich) for 1 h at room temperature in DIG buffer (50 mM NaCl, 300 mM sucrose, 10 mM Tris–Cl, pH 7.4, 3 mM MgCl_2_, 1% (v/v) thiodiglycol, and 0.5% (v/v) Triton X-100) with 1 mM PMSF, 30 mM sodium butyrate and phosphatase inhibitors. An aliquot was saved as the total nuclear fraction. Ammonium sulfate was added slowly to a final concentration of 0.25 M to facilitate the removal of chromatin. The sample was then centrifuged at 9600*g* for 10 min at 4 °C, and the supernatant, termed the ammonium sulfate fraction, was dialyzed against water and saved. The pellet containing the nuclear scaffold was resuspended in 8 M urea. The protein concentration in the four fractions was quantified according to the manufacturer’s instructions using the Pierce BCA Protein Assay kit (Thermo Fisher Scientific). SDS sample loading buffer with or without reducing agent β-mercaptoethanol was added to nuclear scaffold protein fraction. Samples were boiled and analyzed by SDS-PAGE and immunoblotting.

For immunoprecipitation analyses, the nuclear scaffold pellet was resuspended in RIPA buffer (10 mM Tris–HCl pH 8.0, 1% Triton X-100, 0.1% SDS, 0.1% SDC) and sonication was done to fully resuspend the proteins. Anti-UCHL1 antibody (R&D Systems) and control mouse IgG (Millipore) were used, and then, magnetic Dynabeads protein G (Thermo Fisher Scientific) was added. Reducing sample buffer was added to both the IP and IgG beads as well as the input and immunodepleted (ID) fractions that were saved at equal volumes. This was followed by boiling, separation of magnetic beads by a magnet, loading the precipitate in sample buffer on a 12% polyacrylamide gel, SDS-PAGE and immunoblotting.

### UCHL1 knockdown by siRNA transfection

HEK293T cells were transiently transfected with UCHL1 specific ON-TARGET plus siRNA—SMART pool (GE Dharmacon Fisher Scientific). ON-TARGET plus Human GAPDH siRNA was used as positive control and scrambled siRNA as negative control. Transfection was performed using DharmaFECT transfection reagent 1 following the manufacturer’s protocol. Whole cell lysate was isolated after 72 h posttransfection. Immunoblot analysis was performed with the cell lysates with antibodies against UCHL1, RAP1, TRF2 and GAPDH. β-Actin was used as an internal control.

To calculate the knockdown efficiency of UCHL1 siRNA, we performed immunoblot analyses with increasing amounts (0.5–10 µg) of lysate of control and UCHL1 siRNA-transfected cells. Lysates were loaded on a 12% acrylamide gel, and immunoblot analysis was performed using UCHL1-, RAP1- and GAPDH-specific antibodies. The chemiluminescent detection was done with a ChemiDoc gel imaging system, and the protein levels were determined by protein band analysis using Image Lab analysis software (BioRad). To calculate the knockdown efficiency of the UCHL1-siRNA, the intensity of UCHL1 and RAP1 protein bands was normalized with the respective GAPDH intensity (protein for internal control) in control and siRNA-transfected samples. The percentage of remaining UCHL1 and RAP1 in the knockdown samples was calculated by comparing the normalized intensity of UCHL1 and RAP1 proteins with the control.

## Additional files

**Additional file 1.** List of UCHL1 binding sites in DU 145 cells.

**Additional file 2.** List of UCHL1 binding sites within and 5-kb up or downstream genes in DU 145 cells.

**Additional file 3.** GO analysis using software DAVID for the 76 genes corresponding to 191 UCHL1 peaks. The percentage (%) of the input gene count within the term group was calculated. Fold enrichment is the ratio of percentage of user’s genes in a term group versus the percentage of the term genes in human genome (population background). The *p* value was calculated by a modified Fisher’s exact test (EASE score). FDR is the false discovery rate.

**Additional file 4.** In situ PLA images of negative controls used in PLA assays.

**Additional file 5.** Mutant p53 does not associate with UCHL1 binding sites. **a** Expression of p53 in DU 145, PC-3 and HEK293T cells. Ten micrograms of protein (whole cell lysate) was resolved by PAGE and immunoblotted with antibodies against p53 and GAPDH (internal loading control). **b** p53 ChIP qPCR was done for the listed UCHL1 binding sites in PC-3, DU 145 and HEK293T cells. The values are presented as fold enrichment and normalized to mock IgG ChIP and PC-3 p53 ChIP.

**Additional file 6.** UCHL1 may interact with RAP1 as part of a nuclear scaffold complex. Nuclear scaffold lysate from DSP-treated DU 145 cells in RIPA buffer was incubated with an anti-UCHL1 antibody or control IgG. The immunoprecipitate (IP), and equal volumes of lysate (Input) and immunodepleted (ID) fractions were analyzed by immunoblotting with UCHL1 and RAP1 antibodies. Mouse/rabbit Rockland TrueBlot secondary antibodies were used.

## Abbreviations

BJ cells: BJHELTRas^mc^ cells
ChIP: chromatin immunoprecipitation
ITS: interstitial telomeric site
PAGE: two-dimensional polyacrylamide gel electrophoresis
seq: sequencing
RAP1 (TERF2IP): telomeric repeat binding factor 2-interacting protein 1
TRF1/2: telomeric repeat binding factor ½
UCHL1: ubiquitin C-terminal hydrolase isozyme L1
