# Supplementary material for: Ubiquitin C-terminal hydrolase isozyme L1 is associated with shelterin complex at interstitial telomeric sites
Source: Epigenetics Chromatin. 2017 Nov 10;10:54. doi: 10.1186/s13072-017-0160-2 (PMC5681776; doi:10.1186/s13072-017-0160-2)
Supplement: Supplementary file 1 — Additional file 1. List of UCHL1 binding sites in DU 145 cells. [file 13072_2017_160_MOESM1_ESM.pdf]

Additional file 1. List of UCHL1 binding sites in DU 145 cells
* identifies binding sites also detected in HEK293T cells.

Chr	Start	End	Annotation
chr1	10,102	10,425	intergenic*
chr1	18,580,820	18,580,945	intron (IGSF21, intron 2 of 9)
chr1	31,696,859	31,696,959	intron (NKAIN1, intron 1 of 6)
chr1	41,747,585	41,747,691	intergenic*
chr1	91,254,392	91,254,501	intergenic
chr1	111,192,599	111,192,702	intergenic
chr1	119,552,879	119,552,983	intergenic*
chr1	120,176,705	120,176,823	intron (ZNF697, intron 1 of 2)
chr1	155,924,950	155,925,051	intron (ARHGEF2, intron 13 of 21)
chr1	206,214,213	206,214,327	intergenic*
chr1	226,653,327	226,653,428	intergenic
chr1	249,240,084	249,240,278	intergenic*
chr2	10,190	10,292	intergenic
chr2	7,098,990	7,099,118	intron (RNF144A, intron 2 of 8)
chr2	7,358,051	7,358,191	intergenic*
chr2	39,877,354	39,877,465	intergenic
chr2	69,460,719	69,460,845	intron (ANTXR1, intron 17 of 17)
chr2	70,581,611	70,581,731	intergenic
chr2	75,299,579	75,299,730	intron (TACR1, intron 2 of 4)
chr2	107,355,152	107,355,282	intergenic
chr2	114,360,577	114,360,716	intron (DDX11L2, intron 1 of 2)*
chr2	117,835,222	117,835,346	intergenic
chr2	117,835,434	117,835,577	intergenic
chr2	180,728,746	180,728,849	intergenic*
chr2	182,140,505	182,140,611	intron (LOC101927156, intron 3 of 4)
chr2	207,323,305	207,323,419	intron (ADAM23, intron 2 of 25)
chr2	222,928,104	222,928,212	intergenic
chr2	227,330,513	227,330,626	intergenic
chr2	231,325,038	231,325,137	intron (SP100, intron 7 of 13)
chr2	239,392,457	239,392,644	intergenic
chr2	241,553,483	241,553,609	intron (GPR35, intron 2 of 5)*
chr3	9,736,179	9,736,298	intron (MTMR14, intron 17 of 17)
chr3	61,398,388	61,398,488	intergenic
chr3	70,260,579	70,260,698	intergenic
chr3	70,260,944	70,261,102	intergenic
chr3	70,380,334	70,380,433	intergenic
chr3	77,059,779	77,059,901	intergenic
chr3	80,637,038	80,637,146	intergenic
chr3	80,741,876	80,741,976	intergenic
chr3	103,441,774	103,441,898	intergenic
chr3	138,609,096	138,609,208	intergenic

chr3	149,352,208	149,352,308	intron (WWTR1, intron 3 of 7)
chr3	159,411,986	159,412,107	intron (SCHIP1, intron 1 of 6)
chr3	197,900,087	197,900,374	intron (FAM157A, intron 6 of 6)*
chr3	197,901,288	197,901,414	intron (FAM157A, intron 6 of 6)*
chr4	10,052	10,185	intergenic*
chr4	698,901	699,003	promoter-TSS (PCGF3)
chr4	8,349,071	8,349,178	intergenic
chr4	58,205,308	58,205,419	intergenic
chr4	66,920,165	66,920,274	intergenic
chr4	85,754,790	85,754,916	intron (WDFY3, intron 7 of 67)
chr4	137,159,384	137,159,488	intergenic
chr5	10,429	10,529	intergenic*
chr5	11,531	11,631	intergenic
chr5	2,290,931	2,291,031	intergenic
chr5	30,210,518	30,210,639	intergenic
chr5	82,811,907	82,812,020	intron (VCAN, intron 6 of 12)*
chr5	175,233,384	175,233,484	intron (CPLX2, intron 1 of 4)
chr6	147,601	147,760	intron (LINC00266-3, intron 1 of 2)
chr6	1,114,942	1,115,063	intergenic*
chr6	38,912,686	38,912,816	intron (DNAH8, intron 79 of 92)*
chr6	67,465,869	67,466,010	intergenic
chr6	92,453,045	92,453,166	intergenic*
chr6	101,107,388	101,107,495	intron (ASCC3, intron 16 of 41)
chr6	126,072,971	126,073,077	exon (HEY2, exon 2 of 5)
chr6	129,173,352	129,173,456	intergenic
chr6	161,866,788	161,866,892	intron (PARK2, intron 8 of 10)
chr7	10,064	10,203	intergenic*
chr7	16,372	16,503	intergenic
chr7	18,435,671	18,435,782	intron (HDAC9, intron 2 of 12)
chr7	87,177,492	87,177,603	intron (ABCB1, intron 15 of 28)
chr7	127,345,589	127,345,750	intron (SND1, intron 8 of 23)
chr7	127,905,656	127,905,755	intergenic
chr8	13,053,219	13,053,327	intron (DLC1, intron 5 of 17)
chr8	58,877,751	58,877,855	intergenic
chr8	83,014,192	83,014,295	intergenic
chr8	108,091,546	108,091,657	intergenic*
chr9	10,007	10,127	intergenic
chr9	10,153	10,269	intergenic*
chr9	10,288	10,403	intergenic*
chr9	2,824,012	2,824,113	intron (KIAA0020, intron 11 of 17)
chr9	13,958,190	13,958,291	intergenic
chr9	71,880,138	71,880,252	intergenic*
chr9	132,927,632	132,927,745	intergenic*
chr9	137,102,158	137,102,287	intergenic*

chr9	141,023,497	141,023,619	intergenic*	
chr9	141,054,226	141,054,361	intron (TUBBP5, intron 1 of 4)	
chr10	3,986,143	3,986,281	intergenic*	
chr10	4,111,307	4,111,412	intron (LOC101927964, intron 1 of 3)	
chr10	8,510,383	8,510,507	intergenic	
chr10	82,460,299	82,460,406	intergenic	
chr10	102,410,829	102,410,931	intergenic	
chr10	135,524,434	135,524,539	intergenic	
chr11	175,513	175,852	intergenic*	
chr11	55,277,534	55,277,634	intergenic	
chr11	62,795,644	62,795,747	intergenic*	
chr11	74,412,435	74,412,591	intron (CHRDL2, intron 9 of 10)*	
chr11	75,199,635	75,199,745	intron (GDPD5, intron 2 of 16)	
chr11	80,597,901	80,598,033	intergenic*	
chr11	104,636,730	104,636,854	intergenic	
chr11	124,403,456	124,403,570	intergenic	
chr11	129,818,840	129,818,949	intron (PRDM10, intron 4 of 20)*	
chr12	95,286	95,458	intergenic*	
chr12	22,393,946	22,394,047	intron (ST8SIA1, intron 3 of 3)	
chr12	32,096,846	32,096,945	intergenic	
chr12	55,725,773	55,725,886	exon (OR6C3, exon 1 of 1)	
chr12	58,273,051	58,273,151	intergenic	
chr12	59,940,877	59,941,008	intergenic*	
chr12	76,894,996	76,895,115	intron (OSBPL8, intron 1 of 22)	
chr12	98,382,746	98,382,873	intergenic*	
chr12	98,382,920	98,383,127	intergenic	
chr12	98,383,357	98,383,481	intergenic	
chr12	131,419,416	131,419,527	intergenic	
chr13	27,932,178	27,932,309	intergenic	
chr13	46,106,984	46,107,090	intron (COG3, intron 22 of 22)	
chr13	49,635,426	49,635,553	intron (FNDC3A, intron 2 of 25)	
chr13	97,635,584	97,635,707	intron (LINC00359, intron 1 of 2)	
chr14	70,391,447	70,391,546	intron (SMOC1, intron 1 of 11)	
chr15	42,243,142	42,243,265	intron (EHD4, intron 2 of 5)	
chr15	60,167,164	60,167,265	intergenic	
chr15	62,670,353	62,670,452	intergenic	
chr15	64,368,837	64,368,962	intron (FAM96A, intron 2 of 2)	
chr15	79,351,561	79,351,664	intron (RASGRF1, intron 2 of 27)	
chr16	59,970	60,077	intergenic	
chr16	25,771,380	25,771,526	intron (HS3ST4, intron 1 of 1)*	
chr16	75,368,040	75,368,166	intron (CFDP1, intron 5 of 6)*	
chr16	76,150,924	76,151,043	intergenic	
chr16	87,558,042	87,558,239	intergenic	
chr16	88,946,595	88,946,716	intron (CBFA2T3, intron 9 of 10)	

chr17	8,405,816	8,405,930	intron (MYH10, intron 27 of 41)
chr17	35,592,381	35,592,508	intron (ACACA, intron 22 of 53)
chr17	58,276,692	58,276,826	intron (USP32, intron 26 of 33)
chr17	81,194,953	81,195,078	intergenic
chr18	10,273	10,379	intergenic*
chr18	63,654	63,788	intergenic*
chr18	789,532	789,660	intron (YES1, intron 1 of 11)
chr18	33,955,247	33,955,397	intron (FHOD3, intron 3 of 23)
chr18	63,244,084	63,244,184	intergenic
chr18	73,244,644	73,244,748	intergenic
chr18	78,016,220	78,016,343	intergenic*
chr18	78,016,665	78,016,791	intergenic
chr19	245,539	245,667	intergenic
chr19	15,086,547	15,086,739	intron (SLC1A6, intron 1 of 8)
chr19	28,798,510	28,798,636	intergenic*
chr19	37,105,806	37,105,910	intron (ZNF382, intron 4 of 4)
chr19	46,746,583	46,746,689	intron (RNU6-66P, intron 3 of 3)
chr19	59,097,963	59,098,094	intergenic*
chr20	10,172,151	10,172,266	intron (SNAP25-AS1, intron 2 of 4)*
chr20	18,676,094	18,676,206	intron (LOC101929526, intron 1 of 3)
chr20	19,049,972	19,050,105	intergenic
chr20	23,541,646	23,541,747	intergenic
chr20	39,231,761	39,231,880	intergenic
chr20	39,602,595	39,602,728	intergenic*
chr20	59,071,577	59,071,678	intergenic
chr20	62,918,067	62,918,182	intergenic*
chr20	62,918,353	62,918,469	intergenic*
chr20	62,918,583	62,918,739	intergenic*
chr21	36,085,122	36,085,247	intron (CLIC6, intron 5 of 5)*
chr21	45,851,799	45,851,900	intron (TRPM2, intron 27 of 31)
chr22	42,404,062	42,404,170	intron (WBP2NL, intron 1 of 5)
chr22	43,261,776	43,261,881	intergenic*
chr22	45,022,364	45,022,483	intergenic*
chrX	59,970	60,070	intergenic
chrX	12,641,128	12,641,282	intron (FRMPD4, intron 4 of 16)
chrX	28,407,522	28,407,621	intergenic
chrX	31,950,125	31,950,224	intron (DMD, intron 3 of 35)
chrX	48,240,826	48,240,940	intergenic
chrX	48,273,407	48,273,534	intergenic
chrX	73,014,068	73,014,193	exon (TSIX, exon 1 of 1)
chrX	107,154,000	107,154,101	intron (MID2, intron 5 of 9)
chrX	117,779,961	117,780,084	intron (DOCK11, intron 40 of 52)
chrX	120,922,260	120,922,378	intergenic
chrX	133,459,436	133,459,538	intergenic

chrX	138,778,445	138,778,592	intron (MCF2, intron 1 of 28)
chrX	143,436,649	143,436,761	intergenic
chrX	145,811,931	145,812,078	intergenic*
chrX	153,762,529	153,762,631	exon (G6PD, exon 6 of 13)
chrX	155,259,754	155,259,860	intergenic
chrX	155,260,233	155,260,350	intergenic
chrY	9,972	10,102	intergenic*
chrY	768,500	768,605	intergenic
chrY	1,765,122	1,765,240	intergenic
chrY	3,131,474	3,131,607	intergenic
chrY	7,643,522	7,643,622	intergenic
chrY	7,717,580	7,717,683	intergenic
chrY	8,125,053	8,125,170	intergenic
chrY	9,030,369	9,030,505	intergenic*
chrY	17,275,764	17,275,865	intergenic
chrY	20,648,538	20,648,650	intergenic*
chrY	20,995,528	20,995,630	intergenic*
chrY	59,313,103	59,313,202	intergenic
