# Supplementary material for: Ubiquitin C-terminal hydrolase isozyme L1 is associated with shelterin complex at interstitial telomeric sites
Source: Epigenetics Chromatin. 2017 Nov 10;10:54. doi: 10.1186/s13072-017-0160-2 (PMC5681776; doi:10.1186/s13072-017-0160-2)
Supplement: Supplementary file 2 — Additional file 2. List of UCHL1 binding sites within and 5-kb up or downstream genes in DU 145 cells. [file 13072_2017_160_MOESM2_ESM.pdf]

Additional file 2. List of UCHL1 binding sites within and 5 kb up or downstream genes in DU 145 cells. * identifies binding sites also detected in HEK293T cells. ** Expression levels in DU 145 cells were extracted from NCBI GEO GSE71756 and GSE71208.

Chr	Peak Location			Expression level**
	Start	End	Gene name	
chr1	10,102	10,425	intergenic, upstream of DDX11L1*	-
chr1	18,580,820	18,580,945	intron (IGSF21, intron 2 of 9)	-
chr1	31,696,859	31,696,959	intron (NKAIN1, intron 1 of 6)	-
chr1	120,176,705	120,176,823	intron (ZNF697, intron 1 of 2)	+
chr1	155,924,950	155,925,051	intron (ARHGEF2, intron 13 of 21)	+++
chr2	7,098,990	7,099,118	intron (RNF144A, intron 2 of 8)	++
chr2	69,460,719	69,460,845	intron (ANTXR1, intron 17 of 17)	+++
chr2	75,299,579	75,299,730	intron (TACR1, intron 2 of 4)	-
chr2	114,360,577	114,360,716	intron (DDX11L2, intron 1 of 2)*	-
chr2	180,728,746	180,728,849	intergenic, upstream of ZNF385B*	-
chr2	182,140,505	182,140,611	intron (LOC101927156, intron 3 of 4)	NA
chr2	207,323,305	207,323,419	intron (ADAM23, intron 2 of 25)	++
chr2	231,325,038	231,325,137	intron (SP100, intron 7 of 13)	++
chr2	241,553,483	241,553,609	intron (GPR35, intron 2 of 5)*	-
chr3	9,736,179	9,736,298	intron (MTMR14, intron 17 of 17)	++
chr3	149,352,208	149,352,308	intron (WWTR1, intron 3 of 7)	+++
chr3	159,411,986	159,412,107	intron (SCHIP1, intron 1 of 6)	++/-
chr3	197,900,087	197,900,374	intron (FAM157A, intron 6 of 6)*	-
chr3	197,901,288	197,901,414	intron (FAM157A, intron 6 of 6)*	-
chr4	698,901	699,003	promoter-TSS (PCGF3)	++
chr4	85,754,790	85,754,916	intron (WDFY3, intron 7 of 67)	++
chr5	82,811,907	82,812,020	intron (VCAN, intron 6 of 12)*	-
chr5	175,233,384	175,233,484	intron (CPLX2, intron 1 of 4)	-
chr6	147,601	147,760	intron (LINC00266-3, intron 1 of 2	NA
chr6	38,912,686	38,912,816	intron (DNAH8, intron 79 of 92)	-
chr6	101,107,388	101,107,495	intron (ASCC3, intron 16 of 41)	++/+++
chr6	126,072,971	126,073,077	exon (HEY2, exon 2 of 5)	-
chr6	161,866,788	161,866,892	intron (PARK2, intron 8 of 10)	-
chr7	18,435,671	18,435,782	intron (HDAC9, intron 2 of 12)	+
chr7	87,177,492	87,177,603	intron (ABCB1, intron 15 of 28)	-
chr7	127,345,589	127,345,750	intron (SND1, intron 8 of 23)	+++
chr8	13,053,219	13,053,327	intron (DLC1, intron 5 of 17)	++
chr9	10,007	10,127	intergenic, upstream of DDX11L5*	NA
chr9	10,153	10,269	intergenic, upstream of DDX11L5*	NA
chr9	10,288	10,403	intergenic, upstream of DDX11L5*	NA
chr9	2,824,012	2,824,113	intron (KIAA0020, intron 11 of 17)	++
chr9	141,054,226	141,054,361	intron (TUBBP5, intron 1 of 4)*	+
chr10	4,111,307	4,111,412	intron (LOC101927964, intron 1 of 3)	NA
chr11	74,412,435	74,412,591	intron (CHRDL2, intron 9 of 10)	-

chr11	75,199,635	75,199,745	intron (GDPD5, intron 2 of 16)	+
chr11	129,818,840	129,818,949	intron (PRDM10, intron 4 of 20)	++
chr12	22,393,946	22,394,047	intron (ST8SIA1, intron 3 of 3)	-
chr12	55,725,773	55,725,886	exon (OR6C3, exon 1 of 1)	-
chr12	76,894,996	76,895,115	intron (OSBPL8, intron 1 of 22)	
chr13	46,106,984	46,107,090	intron (COG3, intron 22 of 22)	+++
chr13	49,635,426	49,635,553	intron (FNDC3A, intron 2 of 25)	+++
chr13	97,635,584	97,635,707	intron (LINC00359, intron 1 of 2)	NA
chr14	70,391,447	70,391,546	intron (SMOC1, intron 1 of 11)	+
chr15	42,243,142	42,243,265	intron (EHD4, intron 2 of 5)	+++
chr15	64,368,837	64,368,962	intron (FAM96A, intron 2 of 2)	++
chr15	79,351,561	79,351,664	intron (RASGRF1, intron 2 of 27)	-
chr16	59,970	60,077	intergenic, upstream of DDX11L10	-
chr16	25,771,380	25,771,526	intron (HS3ST4, intron 1 of 1)*	-
chr16	75,368,040	75,368,166	intron (CFDP1, intron 5 of 6)	++
chr16	88,946,595	88,946,716	intron (CBFA2T3, intron 9 of 10)	-
chr17	8,405,816	8,405,930	intron (MYH10, intron 27 of 41)	+++
chr17	35,592,381	35,592,508	intron (ACACA, intron 22 of 53)	+++
chr17	58,276,692	58,276,826	intron (USP32, intron 26 of 33)	+++
chr18	10,273	10,379	intergenic, upstream of LOC102723376*	NA
chr18	789,532	789,660	intron (YES1, intron 1 of 11)	+++
chr18	33,955,247	33,955,397	intron (FHOD3, intron 3 of 23)	+++
chr19	15,086,547	15,086,739	intron (SLC1A6, intron 1 of 8)	-
chr19	37,105,806	37,105,910	intron (ZNF382, intron 4 of 4)	++
chr19	46,746,583	46,746,689	intron (RNU6-66P, intron 3 of 3)	NA
chr20	10,172,151	10,172,266	intron (SNAP25-AS1, intron 2 of 4)*	NA
chr20	18,676,094	18,676,206	intron (LOC101929526, intron 1 of 3)	NA
chr20	62,918,067	62,918,182	intergenic, upstream of LINC00266-1*	-
chr20	62,918,353	62,918,469	intergenic, upstream of LINC00266-1*	-
chr20	62,918,583	62,918,739	intergenic, upstream of LINC00266-1*	-
chr21	36,085,122	36,085,247	intron (CLIC6, intron 5 of 5)	-
chr21	45,851,799	45,851,900	intron (TRPM2, intron 27 of 31)	+
chr22	42,404,062	42,404,170	intron (WBP2NL, intron 1 of 5)	-
chr22	45,022,364	45,022,483	intergenic, upstream of LINC00229*	-
chrX	12,641,128	12,641,282	intron (FRMPD4, intron 4 of 16)	-
chrX	31,950,125	31,950,224	intron (DMD, intron 3 of 35)	-
chrX	48,240,826	48,240,940	intergenic, upstream of SSX4B	NA
chrX	73,014,068	73,014,193	exon (TSIX, exon 1 of 1)	+
chrX	107,154,000	107,154,101	intron (MID2, intron 5 of 9)	+
chrX	117,779,961	117,780,084	intron (DOCK11, intron 40 of 52)	++
chrX	138,778,445	138,778,592	intron (MCF2, intron 1 of 28)	-
chrX	153,762,529	153,762,631	exon G6PD, exon 6 of 13	+++
