# Supplementary material for: Ubiquitin C-terminal hydrolase isozyme L1 is associated with shelterin complex at interstitial telomeric sites
Source: Epigenetics Chromatin. 2017 Nov 10;10:54. doi: 10.1186/s13072-017-0160-2 (PMC5681776; doi:10.1186/s13072-017-0160-2)
Supplement: Supplementary file 3 — Additional file 3. GO analysis using software DAVID for the 76 genes corresponding to 191 UCHL1 peaks. The percentage (%) of the input gene count within the term group was calculated. Fold enrichment is the ratio of percentage of user’s genes in a term group versus the percentage of the term genes in human genome (population background). The p value was calculated by a modified Fisher’s exact test (EASE score). FDR is the false discovery rate. [file 13072_2017_160_MOESM3_ESM.pdf]

Additional file 3. GO analysis using software DAVID for the 76 genes corresponding to 191 UCHL1 peaks. The percentage (%) of the input gene Count within the term group was calculated. Fold enrichment is the ratio of percentage of user's genes in a term group versus the percentage of the term genes in human genome (population background). The pValue was calculated by a modified Fisher's exact test (EASE score). FDR is the false discovery rate.

Annotatio	Enrichment Score: 1.3890395271336358						
Category	Term	Count	%	PValue	Genes	Fold Enric	FDR
GOTERM_	GO:000508	4	4.651163	0.002845	ARHGEF2, RASGRF1, MCF2, DOCK11	13.9196	3.408792
GOTERM_	GO:003502	4	4.651163	0.003487	DLC1, ARHGEF2, RASGRF1, MCF2	12.95679	4.979386
GOTERM_	GO:000508	4	4.651163	0.009333	ARHGEF2, RASGRF1, MCF2, DOCK11	9.083132	10.78894
GOTERM_	GO:005105	3	3.488372	0.090137	DLC1, ARHGEF2, MCF2	5.874067	74.87376
GOTERM_	GO:004354	5	5.813953	0.161862	DLC1, ARHGEF2, RASGRF1, MCF2, DOCK11	2.321903	92.43727
GOTERM_	GO:000562	7	8.139535	0.312177	ARHGEF2, RASGRF1, MCF2, ASCC3, DOCK11, ZN	1.496434	98.76366
GOTERM_	GO:003555	3	3.488372	0.448554	ARHGEF2, RASGRF1, MCF2	1.953164	99.9834
Annotatio	Enrichment Score: 0.8981414542411897						
Category	Term	Count	%	PValue	Genes	Fold Enric	FDR
GOTERM_	GO:000371	4	4.651163	0.038614	SP100, CBFA2T3, HDAC9, WWTR1	5.27985	38.08817
GOTERM_	GO:000012	7	8.139535	0.052451	AHRR, SP100, HEY2, PARK2, HDAC9, WWTR1, ZI	2.550868	54.51644
GOTERM_	GO:000813	4	4.651163	0.086482	ARHGEF2, SP100, HEY2, HDAC9	3.773977	66.75594
GOTERM_	GO:004589	4	4.651163	0.288138	SP100, HEY2, CBFA2T3, HDAC9	2.103206	99.30552
GOTERM_	GO:000565	10	11.62791	0.640128	COG3, FAM96A, SP100, ASCC3, HEY2, SKAP2, CI	1.022809	99.99938
Annotatio	Enrichment Score: 0.8163206206093618						
Category	Term	Count	%	PValue	Genes	Fold Enric	FDR
GOTERM_	GO:000635	11	12.7907	0.052378	AHRR, PCGF3, ASCC3, DMD, SND1, ZNF697, PRI	1.918966	54.46484
GOTERM_	GO:000635	13	15.11628	0.057523	SP100, PARK2, CBFA2T3, AHRR, PCGF3, ASCC3,	1.744693	57.94932
GOTERM_	GO:000563	22	25.5814	0.306658	DLC1, FAM96A, SP100, PARK2, WWTR1, CBFA2T	1.156879	98.64205
GOTERM_	GO:000367	7	8.139535	0.587586	AHRR, SP100, ZNF697, PRDM10, HEY2, ZBTB5, R	1.12047	99.99793
Annotatio	Enrichment Score: 0.7441553492464336						
Category	Term	Count	%	PValue	Genes	Fold Enric	FDR
GOTERM_	GO:000012	7	8.139535	0.052451	AHRR, SP100, HEY2, PARK2, HDAC9, WWTR1, ZI	2.550868	54.51644
GOTERM_	GO:004594	7	8.139535	0.161019	ARHGEF2, AHRR, HEY2, PARK2, YES1, WWTR1, Z	1.872197	92.32523
GOTERM_	GO:000370	4	4.651163	0.693265	HEY2, PARK2, CBFA2T3, ZNF382	1.115306	99.99994
