# Supplementary figures and images for: Ubiquitin C-terminal hydrolase isozyme L1 is associated with shelterin complex at interstitial telomeric sites
Source: Epigenetics Chromatin. 2017 Nov 10;10:54. doi: 10.1186/s13072-017-0160-2 (PMC5681776; doi:10.1186/s13072-017-0160-2)

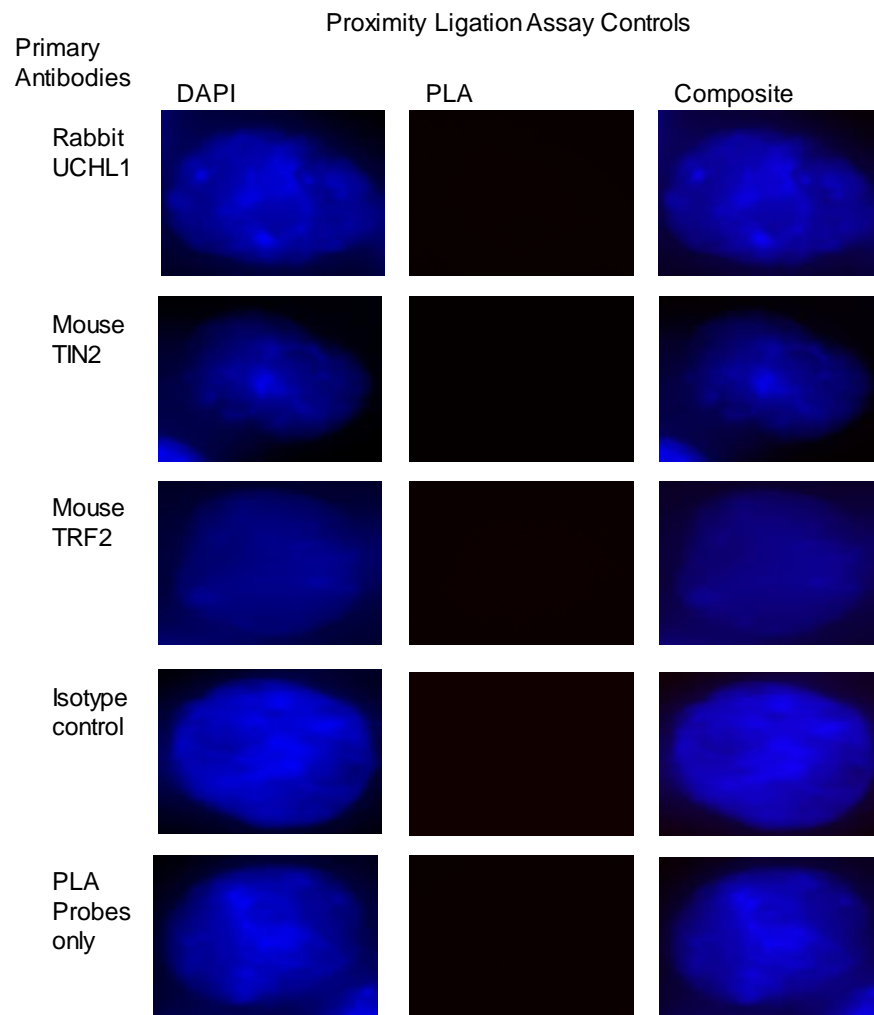

**Additional file 4.** *In situ* PLA images of negative controls used in PLA assays.

Supplement: Supplementary file 4 — Additional file 4. In situ PLA images of negative controls used in PLA assays. [file 13072_2017_160_MOESM4_ESM.pdf]

Additional File 6

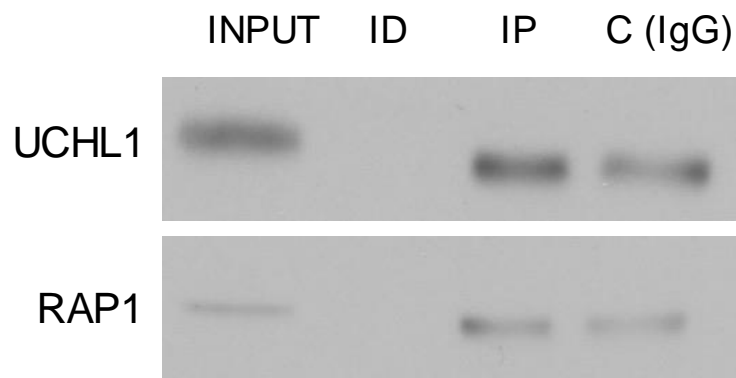

Supplement: Supplementary file 6 — Additional file 6. UCHL1 may interact with RAP1 as part of a nuclear scaffold complex. Nuclear scaffold lysate from DSP-treated DU 145 cells in RIPA buffer was incubated with an anti-UCHL1 antibody or control IgG. The immunoprecipitate (IP), and equal volumes of lysate (Input) and immunodepleted (ID) fractions were analyzed by immunoblotting with UCHL1 and RAP1 antibodies. Mouse/rabbit Rockland TrueBlot secondary antibodies were used. [file 13072_2017_160_MOESM6_ESM.pdf]
