# Supplementary material for: Ubiquitin C-terminal hydrolase isozyme L1 is associated with shelterin complex at interstitial telomeric sites
Source: Epigenetics Chromatin. 2017 Nov 10;10:54. doi: 10.1186/s13072-017-0160-2 (PMC5681776; doi:10.1186/s13072-017-0160-2)
Supplement: Supplementary file 5 — Additional file 5. Mutant p53 does not associate with UCHL1 binding sites. a Expression of p53 in DU 145, PC-3 and HEK293T cells. Ten micrograms of protein (whole cell lysate) was resolved by PAGE and immunoblotted with antibodies against p53 and GAPDH (internal loading control). b p53 ChIP qPCR was done for the listed UCHL1 binding sites in PC-3, DU 145 and HEK293T cells. The values are presented as fold enrichment and normalized to mock IgG ChIP and PC-3 p53 ChIP. [file 13072_2017_160_MOESM5_ESM.pdf]

a b

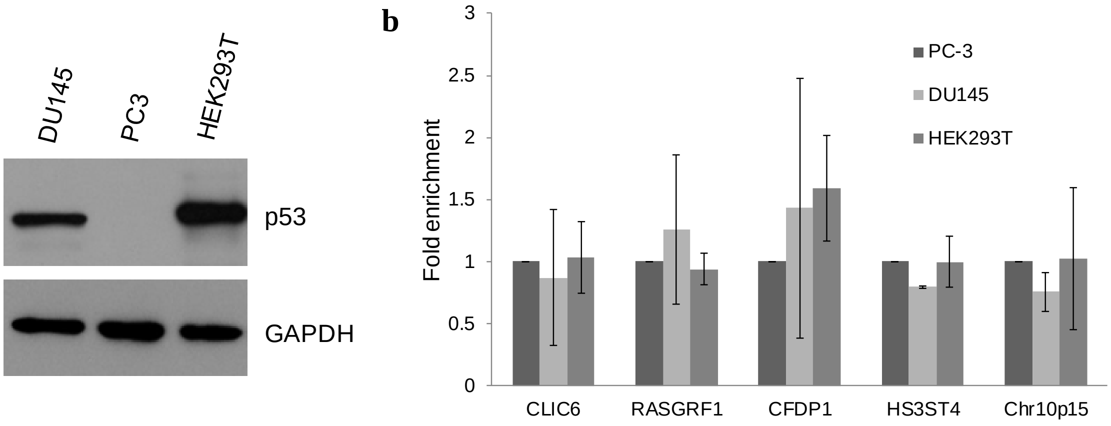

Additional file 5. Mutant p53 does not associate with UCHL1 binding sites. **a** Expression of p53 in DU 145, PC-3 and HEK293T cells. Ten µg of protein (whole cell lysate) were resolved by PAGE and immunoblotted with antibodies against p53 and GAPDH (internal loading control). **b** p53 ChIP qPCR was done for the listed UCHL1 binding sites in PC-3, DU 145 and HEK293T cells. The values are presented as fold enrichment and normalized to mock IgG ChIP and PC-3 p53 ChIP.
